# Associations Between Lactate Thresholds and 2000 m Rowing Ergometer Performance: Implications for Prediction—A Systematic Review

**DOI:** 10.1186/s40798-024-00796-4

**Published:** 2025-02-28

**Authors:** Timothy Kilbey, Eugenio Vecchi, Paulo Salbany, Ashok Handa, Eleanor Stride, Mihir Sheth

**Affiliations:** 1 Department of Engineering Science, Institute of Biomedical Engineering, University of Oxford, Oxford, UK; 2 Nuffield Department of Surgical Sciences, University of Oxford, Oxford, UK; 3 St Catherine’s College, University of Oxford, Oxford, UK

**Keywords:** Physiological markers, Exercise testing, Lactate threshold anaerobic threshold, Sports performance, Exercise threshold

## Abstract

**Background:**

Various exercise thresholds have been evaluated to predict athlete performance. However, a systematic review of the literature assessing the association between lactate-based exercise thresholds and 2000 m rowing ergometer performance is still lacking. These may have utility in the prediction of 2000 m rowing ergometer performance due to the close relationship between metabolic parameters and development of endurance capacity. The aim of the present study is to review and assess the extent, quality, and reliability of lactate-based exercise testing and methodologies in their association with 2000 m rowing ergometer performance, and to discuss the potential implications for performance prediction.

**Methods:**

The systematic review was performed following PRISMA 2020 guidelines. The databases searched were EMBASE, MEDLINE and SPORTDiscus. The initial search took place in July 2022, with an update search performed in September 2023, and again in August 2024. Studies which reported a lactate test and its correlation to 2000 m ergometer performance were included. No meta-analysis was performed.

**Results:**

Twenty-four studies comprising 797 athletes (513 male, 257 female, 27 not stated) met the eligibility criteria for inclusion in the review. The most commonly used testing protocol involved the use of incremental step-tests. A range of exercise intensity parameters, lactate-based exercise thresholds and interpretation methodologies were employed. Of these, the power or velocity at a blood lactate concentration of 4 mmol l^−1^ was the most common test, with correlation coefficients ranging from 0.53 to 0.96 suggesting that 28–92% of the variance in rowing performance can be explained by this metric. Six studies that rated as GOOD on the risk of bias assessment found very strong correlations > 0.85 (*p* < 0.05).

**Conclusions:**

This systematic review found that there is good evidence that the power generated at a blood lactate concentration of 4 mmol l^−1^ correlates strongly to 2000 m rowing ergometer performance and may have useful predictive power. However, the review also identified the varying quality of the available literature, with a variety of parameters, exercise protocols, testing methods, and performance metrics being used to report performance making it difficult to compare results between studies. Other tests such as $\dot{V}{O}_{2}$ at a blood lactate concentration of 4 mmol l^−1^ and power at the initial non-linear inflection blood lactate threshold merit further investigation as the extent and reliability of the available data is currently insufficient to draw firm conclusions.

*Protocol registration:* The protocol was registered on Open Science Framework on 17/11/2022. https://doi.org/10.17605/OSF.IO/D8YCE

**Supplementary Information:**

The online version contains supplementary material available at 10.1186/s40798-024-00796-4.

## Key Points

- The power or velocity that a rower is able to achieve on an ergometer before the concentration of lactate in their blood reaches 4 mmol l^−1^ correlates strongly to their 2000 m ergometer performance.
- The evidence suggests that power generated at the non-linear inflection blood lactate threshold and V̇O_2_ at 4 mmol l^−1^ also have the potential to be useful, but further research needs to be conducted.
- The exercise protocols and performance metrics used to calculate a lactate-based exercise threshold vary between studies. The significant heterogeneity in testing methodology makes it difficult to compare results between studies.
- The reports rarely provide statistical analysis and often conduct studies on a small number of participants making reliability assessment difficult. However, this study found that in aggregate, the findings of power/velocity at 4 mmol l^−1^ are consistent.

**Supplementary Information:**

The online version contains supplementary material available at 10.1186/s40798-024-00796-4.

## Background

For optimal training, athletes and their coaches need to routinely determine their current performance standard. Partaking in a race, however, is a physiological and psychological challenge that interrupts an athlete’s training and thus cannot be performed too frequently. Physiology testing has thus arisen as an invaluable tool for performance prediction for a range of endurance events [1, 2]. Physiological testing also allows for quantitative assessment of the effectiveness of previous training interventions and allows for optimised training regime prescription at set intensities [3]. Conventionally, the maximum rate of oxygen consumption has been used to determine endurance capacity and training status [4, 5], and is indeed positively correlated to rowing ergometer and on-the-water performance in males and females [6–8]. However, there are limitations to this performance metric, not least that it predominantly reflects central circulation and may not discriminate between athletes of similar abilities [9]. Limitations such as these contributed to the development of sub-maximal exercise metrics to assess performance ability [9].

**Fig. 1:**
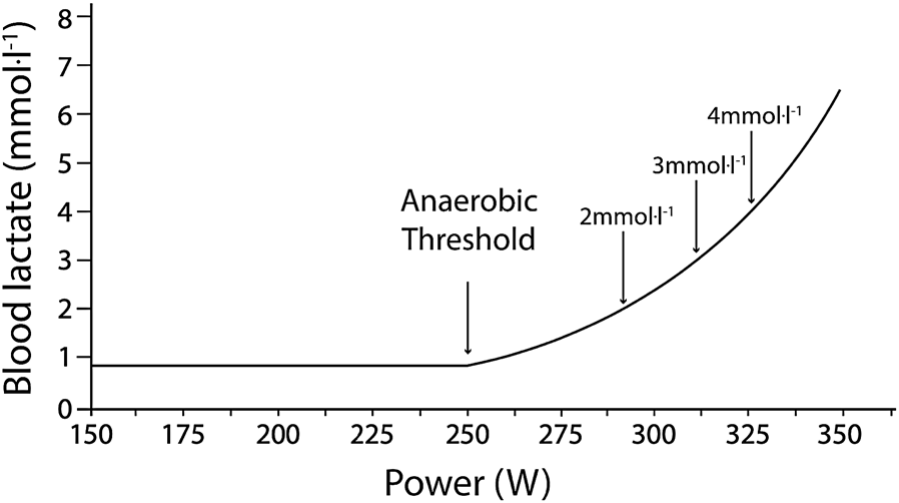
An idealised example of the blood lactate profile, with the various lactate-based exercise thresholds in an athlete undergoing exercise at increasing intensities. A right shift of this graph indicates a greater endurance performance as the athlete is able to perform at a higher exercise intensity before the concentration of lactate in their blood reaches a certain level. Adapted from Arthur Weltman [4]

Historically, exercise intensity has been stratified according to different thresholds which demarcate the point at which the intensity increases from moderate to heavy to severe [10, 11]. Several different thresholds have been described, based on different measurable physiological variables [10]. Of these, lactate-based exercise thresholds have become an important method of assessing endurance sport performance [9]. Assessing these exercise thresholds typically involves an incremental exercise test in which the athlete rows at a gradually increasing intensity, with periodic blood sampling. A graph can then be generated, showing the power (or speed) that a rower can generate before their blood lactate reaches a certain threshold concentration (eg. 4 mmol l^−1^, see idealised example graph Fig. 1). As exercise intensity increases, lactate begins to accumulate in the blood [12]. An athlete with improved endurance capacity will have a rightward shift, being able to generate more power (perform at a greater exercise intensity) before the concentration of lactate in their blood reaches a certain level [13].

One advantage of lactate-based exercise thresholds is that peripheral adaptations such as fibre type and mitochondrial density affect the blood lactate response, suggesting that it may be a useful tool for detecting sport-specific improvements in certain muscle groups. In addition, because endurance events are performed at an intensity that can be maintained for the duration of the exercise, lactate-based exercise thresholds, which determine the standard to which an athlete can perform before lactate reaches a certain level and they begin to fatigue, may be a more meaningful prediction method than maximal oxygen uptake [4]. Furthermore, lactate measurements are easy to collect, with minimal change to the testing environment, although they require specific testing protocols. However, since an elite level 2000 m rowing race lasts only approximately 5:30–7:30 min (depending on boat type and conditions), and 67–88% of the energy contribution of a typical race is provided by aerobic respiration [14], the higher strength demands, and anaerobic capacity of rowers may play a significant role in determining performance.

This systematic review aims to review the extent, quality, and reliability lactate-based exercise testing in their association with 2000 m rowing ergometer performance, and to discuss the potential implications for performance prediction. This review also aims to consolidate the different terminology and methodologies of lactate-based exercise testing.

## Methods

### Registration of Systematic Review Protocol

A systematic review of the literature was performed following the PRISMA 2020 checklist [15]. The protocol was registered with the Open Science Framework in November 2022, (DOI 10.17605/OSF.IO/D8YCE; the full version is included in Online Resource 3).

### Information Sources and Search Strategy

The databases searched were EMBASE, MEDLINE and SPORTDiscus, with the following search strategy. There were minor adaptations to conform to the requirements for different databases:

Search terms:

1. (lactate or anaerobic threshold* or aerobic threshold* or aerobic-anaerobic threshold* or anaerobic–aerobic threshold* or exercise test*)
2. (performance or time or fatigue or exhaustion or result*).mp.
3. (row* or ergo*).mp
4. (2 km or 2000 m or 2,000 m or two-thousand meter or two-thousand metre or two-kilomet* or 2-kilomet* or 2000-m or 2000 m or 2-km).mp
5. 1 and 2 and 3 and 4

In addition, citation searching, keyword searching of the Oxford University Bodleian Library SOLO catalogue, allowed for further identification of relevant studies. The initial search took place in July 2022, with an update search performed in September 2023, and again in August 2024.

### Eligibility Criteria

The eligibility criteria were initially decided by the research team. They were then modified slightly just after publication of the protocol based on a preliminary search to test the validity of the inclusion/exclusion criteria, to ensure the final papers met the aims of this review. The inclusion criteria was broadened from 2 mmol l^−1^ or 4 mmol l^−1^ tests to include any submaximal lactate tests. The exclusion criteria was made more specific to exclude studies that did not report on accuracy and reliability of the lactate scores or the correlation of the lactate scores to the 2000 m rowing performance. The full set of inclusion/exclusion criteria is listed in Table 1.

**Table 1:** Eligibility criteria

Inclusion criteria	Exclusion criteria
Individuals of any age or sex	Studies that fail to report the accuracy or reliability of the lactate scores or its correlation to 2000 m rowing ergometer performance
Amateur or professional rowers	Studies that do not use a rowing ergometer
The use of submaximal lactate tests or maximal blood lactate concentration or maximal plasma lactate concentration	Studies not published in English
Studies on rowing for 2000 m	
Studies published in any year (no date restrictions)	

### Study Selection

Studies were screened for eligibility based on title and abstract. Full texts of eligible studies were then downloaded into Mendeley, any duplicates removed, and independently reviewed by two reviewers (TK, EV) for inclusion. Reasons for exclusion were documented (Online Resource 1). There were no disagreements between the reviewers.

### Data Collection Process

Data were extracted independently by two (TK,EV) reviewers with the use of a standardised form that had been created by three reviewers (TK, EV and MS). Any discrepancies were settled through consultation between the reviewers. The complete list of data points for extraction are listed in Online Resource 2. The protocol was updated to include the mean and standard deviation for the athlete’s weights as opposed to their category as many studies provided the former but not the latter.

### Risk of Bias Assessment

A modified version of the risk of bias assessment developed by Saw et al. [16] was used to conduct a systematic review on the athlete training response, as this was deemed to be the most suitable assessment criterion in the published literature. Modifications involved removing criteria D (training or competition load described) and E (response set on self-report measure described). This is because the majority of included studies are conducted within a few days, so there will be no significant changes in physiology due to training over this period. Similarly, there is no self-reporting involved. Instead, the criterion ‘methodology clearly defined’ was added, with a maximum of two points available for studies that included a detailed methodology that allows the study to be replicated. The entire criteria and scoring is seen in Table 2. If one condition was not clear, the study scored one point, and if two or more conditions were not clear, the study scored zero points in this section. A further binary criterion: ‘statistical test reported’ was also added. Risk of bias assessment was performed independently by two reviewers (TK, EV).

**Table 2:** Risk of Bias Assessment criteria. Adapted From: Saw et al. 2016 [16]

		Scoring		
Criteria	Definition	0	1	2
Peer reviewed	Study Published in a peer-reviewed journal	No	Yes	
Number of participants	Number of participants included in study findings	< 5	5–50	> 50
Population defined	Age, Sex, Sport, Participation level and Experience stated	No	Partly	Yes
Methodology clearly defined	Methodology is clear and detailed enough manner for it to be repeated	No	Partly	Yes
Statistical test reported	A statistical test or significance for each correlation coefficient is discussed	No	Yes	
If Total Score ≥ 7 → Good; If Total Score 4 - 7 → Fair; If Total Score≤ 4 → Poor				

### Data Synthesis

There was significant heterogeneity in the methodology of the incremental tests to determine specific lactate-based exercise thresholds, with differences in initial power, time period of rowing and rest for each step and power increase for each step. There are various terms used for the different exercise thresholds [9]. This study uses the term ‘lactate-based exercise threshold’ to mean any of the specific lactate concentrations or inflection points used in the included studies to correlate to performance. In addition, there was often minimal data regarding the background environment in which both the lactate-based exercise test and 2000 m performance test were performed, with little or no data on feeding status, temperature, humidity, resistance stetting of the ergometer and levels of rest that the athlete had in the week prior to the study. As such, we deemed the heterogeneity of the data too high to generate a meta-analysis.

## Results

### Search Results

**Fig. 2:**
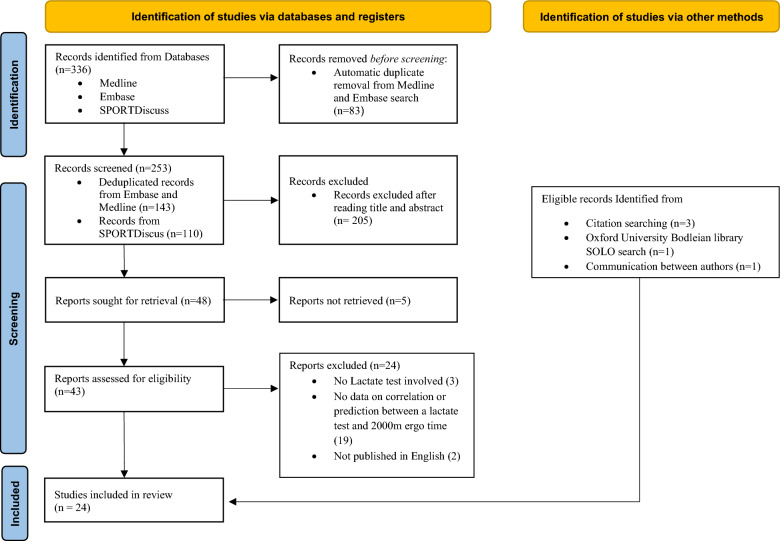
A PRISMA [15] literature search flow chart. n = number of studies. Adapted from Page MJ et al. [15]

The database searches identified 253 records after duplicates were removed. Of these, 43 reports were assessed for eligibility, with 19 being eventually included in the review (18 full studies and one abstract). A further five studies, identified through a combination of citation searching, Oxford University Bodleian Library SOLO searching and consultation with experts were deemed eligible for inclusion. The final update search was competed in July 2024. A summary of the search results and reasons for exclusion are shown in Fig. 2.

### Participants

In total, the studies included 797 participants, of which 513 were male and 257 were female, with the sex of the other 27 participants not stated. 11 studies were exclusively on male rowers [17–27], eight were mixed [6, 28–34], three were exclusively on female rowers [7, 35, 36], and two did not state the sex of the rowers [37, 38]. Both open weight and lightweight rowers were included. 337 participants were elite rowers of at least national standard, with many international standard and world champions rowers included in the studies. There were no recorded novice rowers included in any study, although three studies do not state participant rowing ability [18, 30, 31]. The ‘Development squad’ included in Homer [28] comprised the top ranked, club based athletes not in training as part of the senior international team representing Great Britain at the U23 World Championships and European Rowing Championships [28]. A detailed list of the participants and demographics of each study can be found in Table 3.

**Table 3:** Demographic characteristics of participants in all the studies

Study	Participants	Age (SD)/ Category	Weight (Kg) (SD) / Category	Sex	Level†	Risk of Bias
Bourdin et al. (2004) [17]	54	Total: 22.8 (3.7) LWM: 23 (3.7) OWM: 22.6 (3.7)	Total: 82.4 LWM: 74 (1.8) OWM: 88.6 (5.1)	LWM: 23 OWM: 31	National and international level	GOOD
Bourdin et al. (2017) [7]	70	Total: 21.1(3.3) LWF: 21.9 (3.7) OWF: 20.6 (2.9)	Total: 68.2 (8.3) LW: 60.1 (2.1) OW: 72.8 (6.6)	LWF: 27 OWF: 43	National and International level	GOOD
Brzenczek-Owczarak et al. (2007) [18]	6	15.5 (0.5)	78.4 (10.4)	M	N/S	FAIR
Cosgrove et al. (1999) [19]	13	19.9 (0.6)	73.1 (6.6)	M	Club level	GOOD
Forsyth et al. (2008) [35]	10	33.0 (7.1)	Total: 67.9 (10.5) Individual categories: N/S	LWF: 4 OWF: 6	All regularly use rowing ergometer, 4 compete in rowing events	GOOD
Homer (2014) [28]	53	Senior M: 26.7 (4.2) Senior F: 27.9 (2.8) Dev M: 21.8 (2.2) Dev F: 21.7 (2.0)	Senior M: 96.9 (4.3) Senior F : 76.1 (4.0) Dev M: 93.1 (3.7) Dev F: 79.3 (6.2)	Senior M: 18 Senior F: 14 Dev M: 11 Dev F: 10	National and international level	FAIR
Ingham S et al. (2002)[6]	41	OWM: 25.2 (4.6) LWM: 24.0 (3.5) OWF: 27.2 (4.6) LWF: 26.8 (3.6)	OWM: 92.6 (8.6) LWM: 72.8 (2.2) OWF: 75.4 (5.5) LWF: 59.5 (2.4)	OWM: 19 LWM: 4 OWF: 13 LWF: 5	International level	FAIR
Ingham et al. (2013) [29]	18	23.3 (3.1)	Total: 71.1 (9.3) Individual categories: N/S	OWF: 4 LWF: 4 OWM: 4 LWM: 6	Regional and national Level	FAIR
Jürimäe et al. (1999) [20]	10	18.9 (1.7)	Total: 79.3 (7.3) Individual categories: N/S	OWM: 8 LWM: 2	Experienced	FAIR
Jürimäe et al. (2000) [21]	10	18.9 (1.7)	79.3 (7.3)	M	Experienced	FAIR
Klusiewicz et al. (1991) [37]	15	Juniors: 16.8 (0.8) Seniors: 20.9 (1.0)	Juniors: 81.5 (9.2) Seniors: 76.9 (4.5)	N/S	Club level	POOR
Klusiewicz (1993) [38]	12	24.0 (3.3)	91.2 (6.2)	N/S	International level	FAIR
Klusiewicz A et al. (1994)[30]	46	Younger Junior M: 16.6 (0.9) Older Junior M: 19.1 (0.5) Younger Junior F: 16.9 (1.2)	Younger Junior M: 83.4 (8.6) Older Junior M: 83.6 (6.5) Younger Junior F: 72.2 (7.2)	Younger Junior M: 23 Older Junior M: 10 Younger Junior F: 13	N/S	GOOD
Klusiewicz et al. (1997) [31]	236	M:18.1 (1.4) F: 17.9 (1.5)	M: 84.1 (8.3) F: 71.7 (7.4)	Junior M: 114 Senior M: 54 Junior F: 43 Senior F: 25	N/S	GOOD
Mäestu et al. (1999) [22]	10	18.9 (1.7)	79.3 (7.3)	M	Experienced	GOOD
Messonnier et al. (2005) [23]	21	22 (3.0)	Total: 81.0 (9.0) Individual categories: N/S	LWM: 8 OWM: 13	12 National level 9 International level	GOOD
Nevill et al. (2011) [32]	76	OWM: 23.3 (3.2) LWM: 24.9 (5.0) OWF: 26.1 (4.9) LWF: 25.1 (4.1)	OWM: 94.7 (5.9) LWM: 74.5 (2.8) OWF: 75.5 (5.2) LWF: 59.5 (1.9)	OWF: 21 LWF: 7 OWM: 33 LWM: 15	International level	FAIR
Possamai et al. (2022) [24]	14	26 (13)	81.0 (7.6)	M	Regional and national level	FAIR
Riechman et al. (2002) [36]	12	21.3 (3.6)	67.1 (11.7)	LWF: 6 OWF: 6	Indoor rowing championships participants	GOOD
Schünemann et al. (2023) [33]	10	Total: 19.8 (0.9) M: 19.8 (0.9) F: 19.6 (0.5)	Total: 79.9 (13.3) M: 85.4 (11.2) F: 67.2 (3.8) (6 OW rowers, 4 LW rowers)	M: 7 F: 3	International	GOOD
Turnes et al. (2019) [25]	13	24 (11)	76.4 (6.9)	M	Regional and national level	GOOD
Turnes et al. (2020) [34]	19	25.5 (10.6)	Total: 71.2 (9.1) Individual categories: N/S	M: 16 F: 3	Regional and national level	GOOD
Womack et al. (1992) [26]	18	21.8 (2.6)	87.4 (6.7)	M	Competitive	POOR
Womack et al. (1996) [27]	10	20.4 (1.0)	86.1 (7.3)	M	College level	GOOD

OW(M/F) = Open weight category (Male/Female), LW(M/F) = Lightweight category (Male/Female), M = Male, F = Female, Dev = Development squad, N/S = not stated. †Rowing level as reported by the authors. Where participant age / weight has been stated this was the mean (+ / − standard deviation) when the first measurement was taken

### Testing Methodology

A variety of testing methods and conditions were used in these studies. The discontinuous step-wise incremental rowing technique was the most commonly used testing method (22/25), with the initial power, number of stages and power increase for each stage varying significantly in the different tests. The other testing methods used were continuous step-wise incremental rowing, continuous ramp-wise incremental rowing and a constant rate submaximal test. A detailed list of the techniques and conditions of the test can be found in Table 4. To collect blood to measure blood lactate, eight studies used capillary blood from the fingertips or thumb, 10 studies took blood from the earlobe, two used indwelling venous catheters, one took blood from the toe and three did not specify the location from which the blood was taken.

**Table 4:** Testing conditions and methods for lactate-based exercise testing in each included study

Study	Testing method	Power / conditions	Stages	Location of blood sample
Bourdin et al. (2004) [17]	Discontinuous step-wise incremental rowing	Initial power: Lightweights: 150 W Open weights: 200 W Increment (each stage): 50 W	Duration: 3 min stages Break: 30 s End: Exhaustion	Earlobe
Bourdin et al. (2017) [7]	Discontinuous step-wise incremental rowing	Initial power: Lightweights: 115 W Open weight: 150 W Increment (each stage): 35 W	Duration: 3 min stages Break: 30 s End: Exhaustion	Earlobe
Brzenczek-Owczarak et al. (2007) [18]	Discontinuous step-wise incremental rowing	Initial power: Determined individually Increment (each stage): 30 or 40W (determined individually)	Duration: 3 min stages Break: 1 min End: Exhaustion	Earlobe
Cosgrove (1999) [19]	Discontinuous step-wise incremental rowing	Starting split times between 2:15 and 2:30 depending on participants maximal oxygen uptake Increasing pace by 5 s for each stage	Duration: 5 min stages Break: 30 s End: 5 × stages End: average blood lactate reached 4 mmol l^−1^ or more	Thumb
Forsyth et al. (2008) [35]	Continuous step-wise incremental rowing	Initial power: Individually determined (mean 94.1 W, SD 18.9, range 57.3–119.9 W) Increment (every 3 min): Individually determined (mean 17.5 W, SD 2.4)	Duration: continuous rowing, increasing power every 3 min until exhaustion	Toe
Homer (2014) [28]	Discontinuous step-wise incremental rowing	Initial power: M: 270 W F: 180–200 W Dev Squad: 55% of 2 km average power Increment (each stage): M: 25 W F: 20 W Dev Squad: 5%	Duration: 4 min stages Break: 30 s End: 5 × stages After the last stage, 150 s rest and then 4 min max effort	Capillary blood—location not stated
Ingham et al. (2002) [6]	Discontinuous step-wise incremental rowing	Initial power—not stated Increment (each stage): M: 30 W and 2 strokes/min F: 25 W and 2 strokes/min	Duration: 4 min stages Break: 30 s End: 5 × stages After the last stage, 150 s rest then a 4 min max effort	Earlobe
Ingham et al. (2013) [29]	Discontinuous step-wise incremental rowing	Initial power: N/S Increment (each stage): 25 W and 2 strokes/min	Duration: 4 min Break: 30 s End: 4 stages After the last stage, 150 s rest then a 4 min max effort	Capillary blood—location not stated
	Continuous ramp-wise incremental rowing	Initial power: N/S Increment (every 30 s): 25 W and 2 strokes/min	End: Volitionalexhaustion or power output reduced by 10% of target power for 5 consecutive strokes	
Jürimäe et al. (1999) [20]	Discontinuous step-wise incremental rowing	Initial power: 150 W Increment (each stage): 50 w	Duration: 3 min stages Break: 30 s End: exhaustion	Fingertip
Jürimäe et al. (2000) [21]	Discontinuous step-wise incremental rowing	Initial power: 150 W, Increment (each stage): 50 W	Duration: 3 min stages Break: 30 s End: Once participant max intensity is achieved	Fingertip
Klusiewicz (1991) [37]	Discontinuous step-wise incremental rowing	Initial power: Minors (14–16y): 1.25 W/kg Juniors (16–18 yrs): 1.75 W/kg Seniors (> 19 yrs): 2.25 W/kg Increment (each stage): 0.5 W/kg	Duration: 4 min stages Break: Active rest (exercise to maintain HR of 120–130, time period N/S) End: 3 × stages followed by a 6 min maximal effort	Fingertip
Klusiewicz (1993) [38]	Discontinuous step-wise incremental rowing	Intensity at 50,70 and 85% of average 2 km power output	Duration: 5 min stages Break: 300 s End: 3 × stages	Fingertip
Klusiewicz et al. (1994) [30]	Discontinuous step-wise incremental rowing	Intensity at 50,70 and 85% of average 2 km power output	Duration: 5 min stages Break: 300 s End: 3 × stages	Fingertip
Klusiewicz et al. (1997) [31]	Discontinuous step-wise incremental rowing	Intensity at 50,70 and 85% of average 2 km power output	Duration 5 min stages Break: 5 min End: 3 × stages	Fingertip
Mäestu et al. (1999) [22]	Discontinuous step-wise incremental rowing	Initial power: 150 W Increment (each stage): 50 W	Duration: 3 min stages Break: 30 s End: N/S	Fingertip
Messonnier et al. (2005) [23]	Discontinuous step-wise incremental rowing	Initial power: 150 or 200 W depending on athlete capacity Increment (each stage): 50 W	Duration: 3 min stages Break: 30 s End: Exhaustion	Earlobe
Nevill et al. (2011) [32]	Discontinuous step-wise incremental rowing	Initial power: N/S Increment (each stage): M: 30 W and 2 strokes/min F: 25 W & 2 strokes/min	Duration: 4 min stages Break: 30 s End: 5 × stages After the last stage, 150 s rest then a 4 min max effort	Earlobe
Possamai et al. (2022) [24]	Constant work rate submaximal tests to determine maximal lactate steady state (MLSS)	Initial power: 70% of peak power output Increment (each stage): 5% peak power	Duration 30 min End: 2–4 stages	Earlobe
Riechman et al. (2002) [36]	Discontinuous step-wise incremental rowing	Initial power: 25 W Increment (each stage): 25 W	Duration: 3 min stages Break: 60 s End: Exhaustion	Indwelling catheter—forearm
Schünemann et al. (2023) [33]	Discontinuous step-wise incremental rowing	Initial power: M: 150 W F: 80 W Increment (each stage): M: 50 W F: 40 W	Duration: 4 min stages Break: 30 s End: 5 × stages	Earlobe
Turnes et al. (2019) [25]	Discontinuous step-wise incremental rowing	Initial power: 130 W Increment (each stage): 30 W	Duration: 3 min stages Break: 30 s End: Exhaustion	Earlobe
Turnes et al. (2020) [34]	Discontinuous step-wise incremental rowing	Initial power: M: 130 W F: 75 W Increment (each stage): M: 30 W F: 25 W	Duration 3 min stages Break 30 s End: Exhaustion	Earlobe
Womack et al. (1992) [26]	Discontinuous step-wise incremental rowing	Initial pace: 3.3 m/s Increment: 0.12 m/s	Duration: 3 min stages Break: Not reported End: Not reported	Not stated
Womack et al. (1996) [27]	Discontinuous step-wise incremental rowing	Initial pace: 3.3 m/s Increment: 0.12 m/s	Duration: 3 min stages Break: 60 s End: 5 × stages	Indwelling venous catheter (back of hand or lower forearm)

Here duration is the duration of each stage, break is the resting period for the participant between successive stages, and end is when the trial ends. MLSS = maximal lactate steady state, M = Male, F = Female

### Risk of Bias

Thirteen studies scored a GOOD risk of bias rating, nine scored a FAIR rating and two papers scored POOR (Table 5). No study scored the maximum of eight points available. Six of the studies were not published in peer-reviewed papers (or not clear if peer-reviewed), although this includes the Womack et al. [26] study, which was a conference abstract and was not subject to peer review.

**Table 5:** Risk of bias assessment results

Study	Peer reviewed	Number of participants	Population defined	Methodology clearly defined	Statistical test reported	Overall
Bourdin et al. (2004) [17]	0	2	2	2	1	**7**
Bourdin et al. (2017) [7]	0	2	2	2	1	**7**
Brzenczek-Owczarak et al. (2007) [18]	1	1	1	1	1	**5**
Cosgrove (1999) [19]	1	1	2	2	1	**7**
Forsyth et al. (2008) [35]	1	1	2	2	1	**7**
Homer (2014) [28]	0	2	2	1	1	**6**
Ingham et al. (2002) [6]	1	1	2	1	1	**6**
Ingham et al. (2013) [29]	1	1	2	1	1	**6**
Jürimäe et al. (1999) [20]	1	1	1	2	1	**6**
Jürimäe et al. (2000) [21]	0	1	1	2	1	**5**
Klusiewicz (1991) [37]	1	1	1	1	0	**4**
Klusiewicz (1993) [38]	1	1	1	2	1	**6**
Klusiewicz (1994) [30]	1	1	2	2	1	**7**
Klusiewicz et al. (1997) [31]	1	2	1	2	1	**7**
Mäestu et al. (1999) [22]	1	1	2	2	1	**7**
Messonnier et al. (2005) [23]	1	1	2	2	1	**7**
Nevill et al. (2011) [32]	0	2	2	1	0	**5**
Possamai et al. (2022) [24]	1	1	2	2	0	**6**
Riechman et al. (2002) [36]	1	1	2	2	1	**7**
Schünemann et al. (2023) [33]	1	1	2	2	1	**7**
Turnes et al. (2019) [25]	1	1	2	2	1	**7**
Turnes et al. (2020) [34]	1	1	2	2	1	**7**
Womack (1992) [26]	0	1	1	0	0	**2**
Womack et al. (1996) [27]	1	1	2	2	1	**7**

### Lactate Thresholds

There were a number of different lactate thresholds used. For this paper, we define ‘exercise intensity parameter’ as the independent variable of these papers—the physiological measurement of the athlete during the exercise test. The lactate threshold is the blood lactate concentration ([La-]b) at which the above exercise intensity parameter is measured, which is typically stated in the protocol. The performance metric is the 2000 m time, speed, power or the dependent variable that the studies correlate the lactate-based exercise threshold to.

A lactate threshold of 4 mmol l^−1^, was the most common threshold used (32/69), with other notable thresholds used including the 2 mmol l^−1^ and 3.5 mmol l^−1^ thresholds, as well as the participants individual anaerobic thresholds such as the maximal lactate steady state or point at which there was a non-linear increase in [La-]b (17/69). Alongside a traditional threshold, some studies included correlations for maximal lactate levels or rates of lactate accumulation achieved during or after the exercise test (9/69).

#### Power/Velocity at 4 mmol l^−1^

16 studies involving 630 participants determined the correlation of power/velocity at 4 mmol l^−1^ to 2000 m performance, making it the most common threshold reported. Correlation coefficients ranged from 0.53 to 0.96 (r^2^: 0.28–0.92), indicating that 28–92% of the variance in rowing performance can be explained by this metric. 12 studies found that the total correlation of all their participants had a statistically significant correlation coefficient of r > 0.85 to 2000 m ergometer performance (although two of the studies tested multiple times, sometimes finding a weaker correlation, as detailed in Table 6). Performance was measured relative to time, speed or power, or combinations thereof. Because speed and power are inversly proportional to time, the ergometer is able to use the same input data to calculate each of these metrics. As such, studies that used power or velocity at 4 mmol l^−1^ can be grouped together. Notably, studies that correlated these metrics to 2000 m time as the performance metric may show a negative correlation coefficeint whereas studies that correlate to 2000 m average power or speed as the performance metric may show a positive correlation coefficient.

**Table 6:** Lactate-based exercise tests performed by all the included studies. *indicates p<0.05, ** indicates p<0.01, *** indicates p<0.001, ^i^  indicates no statistical test recorded

Study	Exercise intensity parameter	Lactate threshold	Performance metric (over 2000 m)	Correlation coefficient
Bourdin et al. (2004) [17]	V̇O_2_ as a percentage of the participants maximal oxygen uptake	4 mmol l^−1^	Power	Total: 0.49*** LW: non-significant correlation OW: 0.79***
Bourdin et al. (2017) [7]	Power	4 mmol l^−1^	Power	Total: 0.87*** LW: 0.68*** OW: 0.90***
	Power relative to maximal power output during the incremental test	4 mmol l^−1^		Total: 0.45*** LW: non-significant OW: 0.56***
Brzenczek-Owczarak et al. (2007) [18]	Power	Individual lactate threshold (definition not stated)	Power	Year 1: 0.96** Year 2: 0.90*
Cosgrove et al. (1999) [19]	Lactate maximum 5 min after the participants maximal oxygen uptake test	N/A	Speed	0.58*
	V̇O_2_	Lactate threshold		0.39
	Velocity	Lactate threshold		0.39
	V̇O_2_	4 mmol l^−1^		0.68*
	Velocity	4 mmol l^−1^		0.73**
	Lactate maximum 5 min after 2000 m ergometer maximal effort test	N/A		0.58*
Forsyth et al. (2008) [35]	Power	4 mmol l^−1^	Speed	Mid-follicular phase: 0.82** (CI 0.41–0.96) Mid-luteal phase: 0.82** (CI 0.38–0.96)
Homer (2014) [28]	Power	2 mmol l^−1^	Speed	Total: 0.94** SNR M: 0.44 SNR F: 0.78** DEV M: 0.68* DEV F: 0.41
	Power	4 mmol l^−1^		Total: 0.96** SNR M: 0.54** SNR F: 0.76* DEV M: 0.77** DEV F: 0.66*
Ingham et al. (2002) [6]	Power	2 mmol l^−1^	Speed	Total: 0.92*** F: 0.92*** M: 0.93***
	Power	4 mmol l^−1^		Total: 0.92*** F: 0.89*** M: 0.92***
	Power	Lactate threshold		Total: 0.88*** F: 0.57* M: 0.85***
	Oxygen consumption at as a percentage of maximal oxygen consumption determined by least sum of squares	Lactate threshold		Total: 0.87*** F: 0.69** M: 0.81***
	Oxygen consumption	Lactate threshold		Total: 0.86*** F: 0.52* M: 0.82***
	Maximal blood lactate concentration	N/A		Total: 0.27 F: 0.21 M: 0.31
	Oxygen consumption as a percentage of maximal oxygen consumption	Lactate threshold		Total: 0.12 F: 0.27 M: 0.06
Ingham et al. (2013) [29]	Power	Lactate threshold	Power	0.91*
	Maximum blood lactate concentration achieved in a step-wise incremental test	N/A		0.16
	Maximum blood lactate concentration achieved in a ramp-wise incremental test	N/A		0.52
Jürimäe et al. (1999) [20]	Power	4 mmol l^−1^	Time	− 0.96*
	Power	4 mmol l^−1^	Power	0.95*
Jürimäe et al. (2000) [21]^,γ^	Power	4 mmol l^−1^	Time	− 0.96*
	V̇O_2_	4 mmol l^−1^		− 0.87*
	Lactate at the power of 350 W	N/A		0.96*
Klusiewicz et al. (1991) [37]^,γ^	Power	4 mmol l^−1^	Time	− 0.53^i^
	Power adjusted for bodyweight	4 mmol l^−1^		− 0.81^i^
	Blood lactate concentration after 6-min maximal effort test	N/A		− 0.29^i^
Klusiewicz (1993) [38]	Power	4 mmol l^−1^	Power	Month I: 0.70** Month III: 0.85*** Month V: 0.86*** Month VI: 0.59*
	Blood lactate concentration after 2000 m test	N/A		Month I: − 0.13 Month III: − 0.02 Month V: 0.32 Month VI: 0.27
Klusiewicz et al. (1994) [30]	Power	4 mmol l^−1^	Time	**Tests before and after annual training cycle:** Total before: − 0.93*** after: − 0.86*** Younger M Juniors before: − 0.86*** after: − 0.65*** Older M juniors before: − 0.56 after: − 0.85** Younger F rowers before: − 0.75** after: − 0.60*
	Power adjusted for bodyweight	4 mmol l^−1^		Total before: − 0.74*** after: − 0.74*** Younger M juniors before: − 0.62** after: − 0.37 Older M juniors before: − 0.12 after: − 0.66* Younger F rowers before: − 0.47 after: − 0.24
	Power	4 mmol l^−1^	Power	Total before: 0.94*** after: 0.91*** Younger M juniors before: 0.88*** after: 0.71*** Older M juniors before: 0.58 after: 0.85** Younger F rowers before: 0.77** after: 0.61*
	Power adjusted for bodyweight	4 mmol l^−1^		Total before: 0.76***, after: 0.74*** Younger M Juniors before: 0.61** after: 0.39 Older M juniors before: 0.11 after: 0.57 Younger F rowers before: 0.49 after: 0.26
	Power	4 mmol l^−1^	Power adjusted for bodyweight	Total before: 0.75*** after: 0.72*** Younger M juniors before: 0.36 after: 0.31 Older M juniors before: 0.06 after: 0.21 Younger F junior rowers before: 0.29 after: − 0.03
	Power adjusted for bodyweight	4 mmol l^−1^		Total before: 0.80*** after: 0.81*** Younger M juniors before: 0.67*** after: 0.55** Older M juniors before: 0.62 after: 0.74* Younger F rowers before: 0.81*** after: 0.65*
Klusiewicz et al. (1997) [31]	Power	4 mmol l^−1^	Power	M: 0.795*** F: 0.798***
Mäestu et al. (1999) [22]^,γ^	Power	4 mmol l^−1^	Time	− 0.96*
	V̇O_2_	4 mmol l^−1^		− 0.87*
	Lactate at the power of 350 W	N/A		0.96*
Messonnier et al. (2005) [23]	V̇O_2_	4 mmol l^−1^	Power	0.85***
	V̇O_2_ relative to maximal oxygen uptake	4 mmol l^−1^		0.48
Nevill et al. (2011) [32]	Power	2 mmol l^−1^	Speed	Total: 0.92^i^ F: 0.78^i^ M: 0.77^i^
	Power	3 mmol l^−1^		Total: 0.92^i^ F: 0.82^i^ M: 0.75^i^
	Power	4 mmol l^−1^		Total: 0.91^i^ F: 0.84^i^ M: 0.73^i^
	V̇O_2_	Lactate threshold		Total: 0.92^i^ F: 0.45^i^ M: 0.83^i^
Possamai et al. (2022) [24]	Power	Maximal lactate steady state	Time	− 0.78^i^ (95%CI: − 0.93– − 0.43)
			Speed	0.78^i^ (95%CI: 0.43–0.93)
			Power adjusted for bodyweight	0.66^i^ (95%CI: 0.20–0.88)
Riechman et al. (2002) [36]	Lactate threshold as a percentage of the participants maximal oxygen uptake	N/A	Time	− 0.51
	V̇O2	Lactate threshold		− 0.77**
	Power	Lactate threshold		− 0.82***
	Maximal lactate achieved during the progressive multi-stage test to the participants maximal oxygen uptake	N/A		− 0.37
Schünemann et al. (2023) [33]	Power	4 mmol l^−1^	Time	− 0.72*
	Maximum lactate accumulation rate	N/A		Mixed (n = 8): − 0.67 M only (n = 7): − 0.21
Turnes et al. (2019) (27)	Power	3.5 mmol l^−1^	Power	0.89** (95% CI: 0.71–0.96)
Turnes et al. (2020) [36]	Power	3.5 mmol l^−1^	Power	0.926**
	Power	Lactate threshold		0.916**
Womack et al. (1992) [26]	Velocity	Lactate threshold	Time	− 0.84^i^ (SEE: + / − 0.21)
	V̇O_2_	Lactate threshold		− 0.78^i^ (SEE: + / − 0.24)
	Velocity	2.0 mmol l^−1^		− 0.87^i^ (SEE: + / − 0.19)
	V̇O_2_	2.0 mmol l^−1^		− 0.83^i^ (SEE: + / − 0.22)
	Velocity	2.5 mmol l^−1^		− 0.90^i^ (SEE: + / − 0.17)
	V̇O_2_	2.5 mmol l^−1^		− 0.88^i^ (SEE: + / − 0.19)
	Velocity	4.0 mmol l^−1^		− 0.91^i^ (SEE: + / − 0.16)
	V̇O_2_	4.0 mmol l^−1^		− 0.87^i^ (SEE: + / − 0.19)
Womack et al. (1996) [27]	Velocity	4.0 mmol l^−1^	Time	Pre-training camp: − 0.90* After training camp: − 0.93*
	V̇O_2_	4.0 mmol l^−1^		Pre-training camp: − 0.94* After training camp: − 0.82*

V̇O_2_ = pulmonary oxygen uptake, SEE = standard error of estimate, CI = confidence interval, SNR = Senior, DEV = Development squad, M = Male, F = Female. ^γ^ NOTE—there are considerable similarities between these papers. See discussion for more information

A total of 12 studies (379 participants) included data specifically for male participants whereas six studies (218 participants) recorded female-only data. The 4 mmol l^−1^ threshold showed the highest correlation coefficient magnitude (r = 0.96) of all the thresholds included by the included (full details are in Table 7). In the studies which analysed male and female data separately, the magnitude of correlation coefficients for male (r = 0.54–0.96) and female (r = 0.66–0.90) were within the same range (more details can be seen in Table 8).

**Table 7:** Correlation between power at 4 mmol l^−1^ (W) and 2000 m ergometer performance. *indicates *p* < 0.05, **indicates *p* < 0.01, ***indicates *p* < 0.001. ^i^Indicates no statistical test

Study	Number of participants	Correlation coefficient	2000 m performance metric
Bourdin et al. (2017) [7]	70	Total: 0.87*** LW: 0.68*** OW: 0.90***	Power
Cosgrove et al. (1999) [19]	13	0.734**	Speed
Forsyth et al. (2008) [35]	10	Mid-follicular phase: 0.82** (CI: 0.41–0.96) Mid-luteal phase: 0.82** (CI: 0.38–0.96)	Speed
Homer (2014) [28]	53	Total: 0.96** (SNR M: 0.54**, SNR F: 0.76*; DEV M: 0.77** DEV F: 0.66*)	Speed
Ingham et al. (2002) [6]	41	0.92*** (F: 0.89***, M: 0.92***)	Speed
Jürimäe et al. (1999) [20]	10	− 0.96*	Time
Jürimäe et al. (2000) [21]	10	− 0.96*	Time
Klusiewicz et al. (1991) [37]	15	− 0.53^i^	Time
Klusiewicz et al. (1993) [38]	12	Month I: 0.70* Month III: 0.85*** Month V: 0.86*** Month VI: 0.59*	Power
Klusiewicz (1994) [30],2^α^, 3^ß^	46	Before 1 year training cycle: Total: − 0.93*** Younger M Juniors: 0.86*** Older M Juniors: − 0.56 Younger F Juniors: − 0.75**	Time
		After 1 year training cycle: Total: after − 0.86*** Younger M juniors: − 0.65*** Older M juniors: − 0.85** Younger F: − 0.60*	
Klusiewicz et al. (1997) [31]	236	M: 0.795*** F: 0.798***	Power
Mäestu et al. (1999) [22]	10	− 0.96*	Time
Nevill et al. (2011) [32]	76	0.91ⁱ (Women: 0.84ⁱ, Men: 0.73ⁱ)	Speed
Schünemann et al. (2023) [33]	10	− 0.72*	Time
Womack et al.(1992) [26]	18	− 0.91^i^	Time
Womack et al.(1996) [27]	10	Before training: − 0.90* After training: − 0.93*	Time

OW = Open weight category, LW = Lightweight category. SNR = Senior, DEV = Development squad, CI = confidence interval

^2α^Jürimäe et al. [20] and Klusiewicz et al. [30] and also correlate power at mmol l^−1^ with 2000 m mean power with very similar results, with the slight variation likely due to error in measurement

^3ß^Klusiewicz et al. [30] and [37] also perform these correlations adjusted for bodyweight, which alters the correlation significantly and may be of more use when considering ‘on the water’ rowing performance. See Table 5 for these results

**Table 8:** Correlation between power at 4 mmol l^−1^ (W) and 2000 m ergometer performance disaggregated according to sex. *indicates p<0.05, ** indicates p<0.01, *** indicates p<0.001

Male only correlations		
Study	Number of male participants	4 mmol l^−1^ correlation
Cosgrove et al. (1999) [19]	13 OW	0.734**
Homer (2014) [28]	29 OW (18 senior, 11 junior)	0.54**
		0.77**
Ingham et al. (2002) [6]	23 (19 OW, 4 LW)	0.92***
Jürimäe et al. (1999) [20]	10 (8 OW, 2 LW)	0.95*
Jürimäe et al. (2000) [21]	10	− 0.96*
Klusiewicz et al. (1994) [30]	10 older juniors	Before: − 0.56
		After: − 0.85**
	23 younger juniors	Before: − 0.86***
		After: − 0.65
Klusiewicz et al. (1997) [31]	168 (114 junior, 54 senior)	0.795***
Mäestu et al. (1999) [22]	10	− 0.96*
Nevill et al. (2011) [32]	48 (33 OW, 15 LW)	0.73
Schünemann et al. (2023) [33]	7	− 0.72*
Womack et al. (1992) [26]	18	− 0.91
Womack et al. (1996) [27]	10 OW	Before training: − 0.90*
		After training: − 0.93

Female only correlations		
Study	Number of female participants	4 mmol l^−1^ correlation
Bourdin et al. (2017) [7]	70 (43 OW, 27 LW)	OW: 0.90***
		LW: 0.68***
Forsyth et al. (2008) [35]	10	Mid-follicular phase: 0.82** (CI 0.41–0.96)
		Mid-luteal phase: 0.82** (CI 0.38–0.96)
Homer (2014) [28]	24 OW (14 senior, 10 junior)	Senior: 0.76*
		Junior: 0.66*
Ingham et al. (2002) [6]	18 (13 OW, 5 LW)	0.89**
Klusiewicz et al. (1997) [31]	68 (43 junior, 25 senior)	0.789***
Nevill et al. (2011) [32]	28 (21 OW, 7 LW)	0.84

OW = Open Weight category, LW = Lightweight category

#### $\dot{{\varvec{V}}}{{\varvec{O}}}_{2}$ at 4 mmol l^−1^ Blood Lactate

Seven studies [17, 19, 21–23, 26, 27] comprising a total of 136 male participants included measurements of pulmonary oxygen uptake ($\dot{V}{O}_{2}$) at 4 mmol l^−1^ [La-]b and its correlation to 2000 m performance (Table 9). Correlation coefficients ranged from r = 0.49 to 0.94 (r^2^: 0.24–0.88), indicating that 24–88% of the variance in rowing performance can be explained by this metric.

**Table 9:** Correlation between ${\dot{\text{V}}}{\text{O}}_{2}$ at 4 mmol l^−1^ and 2000 m ergometer performance *indicates *p* < 0.05, ***indicates *p* < 0.001, ^i^indicates no statistical test performed

Study	Number of participants	Correlation coefficient	2000 m performance metric
Bourdin et al. (2004) [17]	54	OWM 0.79***	Power
		LWM Not Sig	
		Total 0.49***	
Cosgrove et al. (1999) [19]	13	0.68*	Speed
Jürimäe et al. (2000) [21]	10	− 0.87*	Time
Mäestu et al. (1999) [22]	10	− 0.87*	Time
Messonnier et al. (2005) [23]	21	0.85***	Power
Womack et al. (1992) [26]	18	− 0.87^i^	Time
Womack et al. (1996) [27]	10	Before training − 0.94*	Time
		After Training − 0.82*	

OWM = Open Weight category Male, LWM = Lightweight category Male

Four studies (48 athletes) showed a correlation coefficient magnitude r > 0.85, however Womack et al. [26] did not report any statistical test and Womack et al. [27] found that the correlation decreased to r = 0.82 after a rowing training camp.

#### Measurements at Other Lactate Thresholds

Sixteen studies investigated the correlation between a measurement at the lactate threshold (sometimes called the anaerobic threshold) and 2000 m performance (Table 10). In addition, one study investigated the power output at the maximal lactate steady state [24]. Eight studies defined the lactate threshold as 4 mmol l^−1^ [La-]b [20–22, 30, 31, 33, 37, 38].The results of these eight studies are thus instead included in the above sections. Two studies defined this threshold as 3.5 mmol l^−1^ [La-]b and found significant (*p* > 0.005), strong correlations between power at this threshold and 2000 m performance. The majority of the remaining studies all used this term to describe an individual anaerobic threshold such as the point as which there is a non-linear increase in [La-]b. Indeed, using power at this threshold, three studies [6, 29, 34] found a strong correlation r > 0.85 (*p* < 0.05) to 2000 m performance. As can be seen in Ingham et al. [6] and Nevill et al. [32] the female correlation coefficients were smaller than both the male and total correlation coefficients, (Ingham et al. [6]: r = 0.57* (female) vs. r = 0.85*** (male) and r = 0.88*** (total) and Nevill et al. [32]: r = 0.45 (female) vs. r = 0.83 (male) and r = 0.92 (total)) irrespective of the type of test conducted.

**Table 10:** Individual lactate thresholds used. *indicates p < 0.05, ** indicates p < 0.01, *** indicates p < 0.001, ^i^ indicates no statistical test recorded.

Study	Number of participants	Exercise intensity parameter	Definition of lactate threshold used	2000 m performance metric	Correlation coefficient
Brzenczek-Owczarak et al. (2007) [18]	6	Power	‘Individual lactate threshold’, definition N/S	Power	Year 1: 0.96** Year 2: 0.90*
Cosgrove et al. (1999) [19]	13	$\dot{V}{\text{O}}_{2}$	The inflection point of the lactate profile which indicated a sharp increase in blood lactate concentration	Speed	0.39
		Velocity			0.39
Ingham et al. (2002) [6]	41	Power	The concentration at which a non-linear increase occurs in [La-]b	Speed	Total: 0.88*** Female: 0.57* Male: 0.85***
		Oxygen consumption as a % of maximal oxygen uptake determined by least sum of squares			Total: 0.87*** Female: 0.69* Male: 0.81***
		Oxygen consumption			Total: 0.86*** Female: 0.52* Male: 0.82***
Ingham et al. (2013) [29]	18	Power	The breakpoint in the profile of [La-]b against V̇O_2_ where a marked and sustained increase in [La-]b (> 1 mmol l^−1^) was observed from baseline	Power	0.91*
Nevill et al. (2011) [32]	76	$\dot{V}{\text{O}}_{2}$	A non- linear increase in [La-]b	Speed	Total: 0.92ⁱ Female: 0.45ⁱ Male: 0.83ⁱ
Possamai et al. (2022) [24]^a^	14	Power	Maximal lactate steady state workload-the highest power output where [La-]b did not increase by > 1 mmol l^−1^ between minutes 10 and 30 of the constant load test	Time	− 0.78^i^
Riechman et al. (2002) [36]	12	Power	The point at which a 1 mmol l^−1^ [La-]b increase above baseline measurements occurs	Time	− 0.82***
		$\dot{V}{\text{O}}_{2}$			− 0.77**
		VO_2_ as a % of maximal oxygen uptake			− 0.51
Turnes et al. (2019) [25]	13	Power	‘anaerobic threshold’ 3.5 mmol l^−1^	Power	0.89** (CI: 0.71–0.96)
Turnes et al. (2020) [34]	19	Power	‘anaerobic threshold’ 3.5 mmol l^−1^	Power	0.926**
			The intensity maintained during the stage prior to which the first sudden and sustained increase in [La-]b above the baseline level was observed		0.916**
Womack et al. (1992) [26]	18	Velocity	Not stated	Time	− 0.84ⁱ (SEE: + / − 0.21)
		$\dot{V}{\text{O}}_{2}$			− 0.78ⁱ (SEE: + / − 0.24)

^*^indicates *p* < 0.05, ** indicates *p* < 0.01, ***indicates *p* < 0.001 ⁱindicates no statistical test performed. V̇O_2_ = pulmonary oxygen uptake, SEE = standard error of estimate, CI = confidence interval Any test that defined ‘lactate threshold’ as the 4 mmol l^−1^ threshold are instead included in the other tables for 4 mmol l^−1^ tests

^a^Possamai also records maximal lactate steady state power output correlating to 2000 m power as 0.78

## Discussion

This systematic review is believed by the authors to be the first of its kind, reviewing the quality, extent, and reliability of studies comparing 2000 m ergometer performance with lactate measurements. It showed that there is a strong body of evidence suggesting that the power at a [La-]b of 4 mmol l^−1^ correlates strongly to 2000 m performance, irrespective of sex. It also showed that there a lack of a standard protocol or best practice resulting in the use of different protocols, variables, definitions, and performance metrics.

The quality of published literature regarding lactate-based exercise threshold testing is variable, as identified by the risk of bias assessment criteria used in this review. 11/24 of the included studies did not score GOOD on the risk of bias, indicating that their results may be less reliable. Similarly, 16/24 studies used small cohorts of less than 20 participants and so individually, their results may be underpowered. However, this systematic review helps aggregate the data. Furthermore, most studies included only the *p*-value or confidence interval / standard error of estimate when reporting their results. Where studies included both of these alongside each other, this enabled more informative conclusions to be drawn regarding the significance and utility of these results. Two studies included *p*-value and confidence interval [25, 35], and one other included a *p*-value and SEE [29]. Two studies provided neither the *p*-value nor the confidence interval / standard error of estimate [32, 37]. There is a lack of statistical analyses in each study, which reduces the reliability of the findings. However, the fact that despite varying methodologies and small sample sizes, power at 4 mmol l^−1^ [La-]b remains well correlated to 2000 m performance, indicates that it may be a reliable marker of performance. However, this is just one variable, and care must be taken in interpretation of the other variables.

In four studies [6, 28, 30, 32], the correlation of the entire cohort was seen to be higher than the correlation of just males, or just females. This phenomenon may relate to the geometry of linear regression analysis. This result is quite common in regression, and it reflects the fact that each new explanatory variable generally gives some additional information about the response variable, so that a model that combines variables from two other models will give a higher coefficient of determination than the latter models. This could reduce the usefulness of this test when comparing athletes of a similar standard and physiological metrics, or when monitoring marginal improvements of an athlete—the key use of lactate-based exercise thresholds.

### Key Findings

#### Power at 4 mmol l^−1^ [La-]b

The results suggest that power at 4 mmol l^−1^correlates strongly to 2000 m ergometer performance, with six studies that rated as GOOD on the risk of bias assessment, and 6 other studies finding very strong correlations r > 0.85(*p* < 0.05), suggesting that this metric explains more than 72% of 2000 m performance. However, sub-group analysis based on weight and experience categories broadly reduced the strength of the correlations. Only one study that was rated as GOOD on the risk of bias assessment included this data for female rowers. Nevertheless, of the available evidence, there does not seem to be an important difference in results depending on sex. In addition, whilst 12/16 studies indicate strong correlations (r > 0.85, r^2^ > 0.72), the total r^2^ value ranges from 0.28 to 0.92, which may be due to the lack of consistency of exercise protocols amongst studies. This metric has the potential to be a good predictor for 2000 m performance, but future prediction modelling studies should include a validation test in human participants.

#### $\dot{V}{O}_{2}$ at 4 mmol l^−1^ [La-]b

The results suggest that $\dot{V}{O}_{2}$ at 4 mmol l^−1^ [La-]b may be a useful predictor of 2000 m performance, with 4/7 included studies finding a very strong correlation (r > 0.85). However, only two of these papers scored GOOD [22, 27], and when they repeated the tests in the trained individuals in the Womack et al*.* study, the correlation fell to below 0.85 [27]. As such, several large-scale studies on different categories of rower are required to confirm the reliability of this measurement and its usefulness in predicting 2000 m performance.

#### Measurements at the Lactate Threshold

Seven studies correlated performance to a lactate threshold other than a fixed lactate value. However, there was a lack of consistency in terminology with the term ‘lactate threshold’ being defined in different ways (Fig. 1 indicates the various lactate thresholds). Even where ‘lactate threshold’ is taken to mean the point at which there is a marked, non-linear inflection, the exact point at which this occurs is subjective. Slight inaccuracies in determining this point could render prediction models unacceptably inaccurate. As such, two studies [6, 29] had two independent reviewers to denote this point, but the number of reviewers is not mentioned in five other studies [18, 19, 32, 34, 36]. Despite this, 3/4 of the studies that use this subjective technique to assess power at the lactate threshold found a correlation to 2000 m performance of a magnitude of r > 0.85 (*p* < 0.05) [6, 29, 34]. Because this exercise threshold is specific to an individual, it is feasible that it could be a more reliable performance predictor than fixed [La-]b thresholds.

### Key Differences

#### Testing Methodology

There are significant differences in the testing methodologies employed by each study. Despite most studies using step-wise incremental testing, there is considerable variation in the initial intensity, rest period, power increment for each step and number of and duration of steps that may have affected the results. Indeed, Bourdin et al. [39] found that varying the interval duration of each step significantly alters the power output calculated at the anaerobic threshold when compared to the power output at the Maximal Lactate Steady State (MLSS). Power output and V̇O_2_ of the rowers were higher during three-, four- and five-minute incremental tests than the measured values at MLSS^1^, suggesting that many of the included studies may not have achieved a lactate steady state. This means that for each rowing step, blood samples may have been taken before the [La-]b reaches equilibrium for that power output, which may result in an overestimated lactate threshold for the rowers. Furthermore, some studies increase the power in each step by 50 W [17, 20–22]. This is a large increase in power and thus it is unlikely to generate a precise indication of the power at which a lactate threshold is crossed.

Most studies do not discuss the environmental conditions or the ergometer resistance setting in which the tests were performed. A 2000 m test is a significant physiological challenge and may be affected by room temperature, humidity, time of day, sleep, and state of mind. In addition, many do not describe the participant’s diet which could significantly affect glycogen levels, especially in lightweight rowers. This may be an important omission, with a 2005 crossover study indicating that fasting plasma lactate levels were significantly lower across multiple power outputs during a stepwise incremental exercise test [40] as compared to when the test was performed in the fed state. Similarly, physiological studies are often performed during a rowing training camp where there is an elevated training load which may lead to glycogen depletion, which has been shown by Carl Foster et al*.* to depress [La-]b during submaximal exercise, leading them to suggest that it may be beneficial to normalize for peak exercise [La-]b to correct for this [41]. However, this technique is not performed in the included studies. Future studies should follow a standardised format that includes the warm-up intensity and period, nutrition of the athletes, temperature of the room, and model and resistance settings of the ergometers.

#### Participants

The studies in this review include a reasonably diverse participant population, with differing rowing categories, ages, biological sex, experience and ability, although all the included studies involved exclusively experienced rowers, typically at an elite standard (as reported by the authors). Using only experienced athletes with effective pacing strategies may have reduced performance variations that may have otherwise affected the results. One potential role of lactate-based exercise thresholds is that they may be able to help to identify potential in novice athletes who do not yet have the correct technique to achieve a representative 2000 m performance. The proportion of slow twitch vs fast twitch fibres differs in endurance and sprint athletes. Fast-twitch fibres operate through glycolytic metabolism and thus produce more lactate than slow twitch metabolism. Thus, lactate-based exercise thresholds have the potential to provide insight on the current make-up of a novice’s muscle make-up without needing a muscle biopsy [42]. However, further research is needed to elucidate whether these physiological differences alter the accuracy and reliability of lactate-based exercise testing.

#### Blood Sampling Techniques

There is no consensus in the literature on the importance of the location of blood sampling [43, 44], however it is likely to be less important than some of the other methodological differences discussed. The timings of blood sampling after the completion of each stage is also not consistently reported, suggesting that there might be some variability. However, a 2009 study reported that the correlation between [La-]b in rowers at 15 s and 45 s was (r = 0.97 (*p* < 0.05)), across a range of different [La-]b values [45], indicating that these differences are unlikely to have affected the conclusion of this review. Nevertheless, the use of indwelling catheters for continual blood sampling (or more frequent sampling from the toe, performed during the rowing action) may increase precision in identifying the lactate threshold.

### Study Limitations

This systematic review has several limitations that may affect the conclusions. Firstly, whilst efforts were made to refine the search terms appropriately, the search terms may not have included every relevant paper available from the selected databases. Similarly, there may be other relevant papers that are not available on these databases, particularly studies that are older and were not uploaded to online databases. Extensive citation searching and consultation with experts in the field mitigated this risk as far as reasonably possible.

In addition, there is a significant risk of publication bias, with strong correlations and novel lactate testing methodology being more likely to be published, especially as many of these studies did not publish in peer-reviewed journals. Furthermore, because many of the studies had small sample sizes and involved multiple tests (not just lactate-based exercise thresholds) that were correlated with performance, it is possible that some of the tests will by chance be strongly correlated to the 2000 m performance, especially in the studies that failed to report statistical significance.

Three of the included studies [20–22] involve 10 participants with the same average age, correlation coefficients and *p* values, and have similar authorship, suggesting that these three data points are perhaps not entirely separate studies. If this is the case, the conclusions of this review are supported by considerably less data, as these three papers provide evidence for a strong correlation of power at 4 mmol l^−1^ and $\dot{V}{O}_{2}$ at 4 mmol l^−1^ [La-]b to 2000 m performance.

Importantly, whilst the data indicate that some lactate-based exercise thresholds may be accurate and useful predictors of 2000 m ergometer performance, this may vary depending on athlete profile and category. This review included data predominantly from high level athletes who will have a relatively stable 2000 m time, but there is no data on novice athletes. Future research is needed to understand how athlete profile and category affects the usefulness of lactate-based exercise thresholds and performance prediction, especially in novice rowers.

Finally, whilst this review assimilated the available data regarding the correlation between lactate-based exercise thresholds and 2000 m rowing performance, enabling the conclusion that some thresholds may be good predictors of performance, actual prediction requires a predicative model to be built and validated. We urge researchers in the field to build on this, building testing predicative models using the most promising thresholds identified in this review.

## Conclusion

This systematic review shows that there is good evidence that the power at a [La-]b of 4 mmol l^−1^ correlates strongly to 2000 m performance on a rowing ergometer. As such, it may be a useful tool for rowing coaches to assess performance on a more regular basis without pushing an athlete to the physical and psychological limit of performing a 2000 m test. However, the evidence that it may be a useful predictor of performance is more limited in categories with less available data. There are no studies involving novice rowers and limited data on lightweight and female rowers. Nevertheless, the available data suggests that power at a [La-]b of 4 mmol l^−1^ remains strongly correlated to 2000 m performance in female athletes.

Furthermore, much of the published literature is not of high quality or reliability A stronger conclusion regarding the accuracy and usefulness of lactate-based exercise thresholds will require future studies that use a pre-registered, clear methodology that reflects the current understanding of lactate production and clearance, especially with regards to stage/step duration and increments in power and rate. These studies should separate the results according to sex and rowing category to allow for optimal analysis of the results. This review has identified that the most promising lactate-based exercise thresholds, which should be the focus of these future studies assessing the predictive power of power and $\dot{V}{O}_{2}$ at a [La-]b of 4 mmol l^−1^, and power/$\dot{V}{\text{O}}_{2}$ at the initial non-linear inflection blood lactate threshold.

## Supplementary Information

**Additional file 1.** Online Resource 1 - Exclusion List.

**Additional file 2.** Online Resource 2 - Data Extraction.

**Additional file 3.** Online Resource 3 - Protocol.

**Additional file 4.** Online Resource 4 - PRISMA Checklist.

## Data Availability

The datasets used and analysed during the current study are available from the corresponding author on reasonable request.

## Abbreviations

$\dot{V}{O}_{2}$: Pulmonary oxygen uptake
[La-]b: Blood lactate concentration
MLSS: Maximal lactate steady state
OW: Open weight rower
LW: Lightweight rower
OWF: Open weight category female rower
LWF: Lightweight category female rower
OWM: Open weight category male rower
LWM: Lightweight category male rower
W: Watts (power)

## Footnotes

1 1 MLSS was determined from at least three 30-min constant intensity tests.
