# Supplementary material for: Associations Between Lactate Thresholds and 2000 m Rowing Ergometer Performance: Implications for Prediction—A Systematic Review
Source: Sports Med Open. 2025 Feb 28;11:21. doi: 10.1186/s40798-024-00796-4 (PMC11871166; doi:10.1186/s40798-024-00796-4)
Supplement: Supplementary file 1 — Additional file 1. Online Resource 1 - Exclusion List. [file 40798_2024_796_MOESM1_ESM.pdf]

Title: Utility of lactate testing for the prediction of 2000m rowing ergometer performance: a systematic review

Journal: Sports Medicine – Open

Author Names: Timothy Kilbey†, Eugenio Vecchi†, Paulo Salbany, Prof. Ashok Handa, Prof. Eleanor Stride, Mihir Sheth*

† These authors contributed equally to this work.

*Correspondence should be addressed to mihir.sheth@nds.ox.ac.uk

Affiliations

Department of Engineering Science, Institute of Biomedical Engineering, University of Oxford, Oxford, U.K

Mihir Sheth, Prof. Eleanor Stride

Nuffield Department of Surgical Sciences, University of Oxford, Oxford, U.K

Prof. Ashok Handa, Paulo Salbany

St Catherine's College, University of Oxford, Oxford, U.K

Timothy Kilbey, Eugenio Vecchi

Reference	Reason for exclusion
Jurimae J, Jurimae T. A comparison of selected anthropometric, metabolic and hormone parameters in lightweight and open-class rowers. Biology of Sport [Internet]. 2002 [cited 2024 Aug 3];19(2):149–61.	Unable to retrieve full text, no data in abstract
Riganas, Christos & Vrabas, Ioannis & Konstantinos, Mandroukas. (2009). Aerobic mechanism relative contribution during a simulated 2000m "all-out" race. Inquiries in Sport & Physical Education.	Published in Greek
Obminski Z, Klusiewicz A, Stupnicki R. Changes in salivary and serum cortisol concentrations in junior athletes following exercises of different intensities. Biology of Sport [Internet]. 1994 [cited 2024 Aug 3];11(1):49–57.	Published in Polish, only a translated document
Klusiewicz A, Faff J, Zdanowicz R. Diagnostic value of indices derived from specific laboratory tests for rowers. Biology of Sport [Internet]. 1999 [cited 2024 Aug 3];16(1):39–50.	Study fails to report the correlation of the lactate score to 2000m rowing ergometer performance
Faff J, Bienko A, Burkhard-Jagodzinska K, Borkowski L. Diagnostic value of indices derived from the Critical Power Test in assessing the anaerobic work capacity of rowers. Biology of Sport [Internet]. 1993 [cited 2024 Aug 3];10(1):9–14.	Unable to retrieve full test, no data in abstract
Mikulic P. Maturation to elite status: a six-year physiological case study of a world champion rowing crew. European Journal of Applied Physiology [Internet]. 2011 Aug [cited 2024 Aug 3];111(9):2363–8.	No lactate test or maximal blood lactate concentration of maximal plasma lactate concentration
Ebert T, Davoren W, Osgood R. Physiological and anthropometrical changes in schoolboy rowers over a competition period. Biology of Sport [Internet]. 2000 [cited 2024 Aug 3];17(3):155–67.	Unable to retrieve full text
Gayer CD. Physiological discriminators of rowing performance in male, club rowers. 1994 [cited 2024 Aug 3];	Unable to retrieve full text
Godfrey R, Ingham S, Pedlar C, Whyte G. The detraining and retraining of an elite rower : a case study. Journal of Science & Medicine in Sport [Internet]. 2005 Sep [cited 2024 Aug 3];8(3):314–20.	No 2000m test
Gillies EM, Bell GJ. The relationship of physical and physiological parameters to 2000m simulated rowing performance. Sports Medicine, Training & Rehabilitation [Internet]. 2000 [cited 2024 Aug 3];9(4):277–88.	No submaximal Lactate Tests or maximal blood lactate concentration or maximal plasma

	lactate concentration
Luo X, Zhang D, Yu W. Uniform Homeostatic Stress Through Individualized Interval Training Facilitates Homogeneous Adaptations Across Rowers With Different Profiles. International Journal of Sports Physiology & Performance [Internet]. 2024 Mar [cited 2024 Aug 3];19(3):232–41.	Unable to retrieve full text
MEDLINE SEARCH	
Thompson KMA, PethickWA, Clarke J, Winegarden A, Johnson E, Coates AM, Burr JF (2024). Blood flow restricted training and time trial performance: a cohort study of world class rowers. Medicine & Science in Sports & Exercise, https://dx.doi.org/10.1249/MSS.0000000000003459	Study fails to report the correlation of the lactate score to 2000m rowing ergometer performance
PossamaiL, Borszcz F, de Aguiar R, Lucas R, Turnes T. Comparison of NIRS exercise intensity thresholds with maximal lactate steady state, critical power and rowing performance. Biology of Sport. 2024;41(2):123-130.doi:10.5114/biolsport.2024.129486.	Study fails to report the correlation of the lactate score to 2000m rowing ergometer performance
Possamai, L. T., deAguiar, R. A., Borszcz, F. K., do Nascimento Salvador, P. C., de Lucas, R. D.,& Turnes, T. (2022). Muscle Oxidative Capacity in Vivo Is Associated With Physiological Parameters in Trained Rowers. Research Quarterly for Exercise and Sport, 94(4), 1020–1027. https://doi.org/10.1080/02701367.2022.2100862	Study fails to report the correlation of the lactate score to 2000m rowing ergometer performance
Turnes, T., deAguiar, R.A., de Oliveira Cruz, R.S. et al. Impact of ischaemia–reperfusion cycles during ischaemic preconditioning on 2000-m rowing ergometer performance. Eur J Appl Physiol 118, 1599–1607 (2018). https://doi.org/10.1007/s00421-018-3891-2	Study fails to report the correlation of the lactate score to 2000m rowing ergometer performance
Ní Chéilleachair NJ,Harrison AJ, Warrington GD. HIIT enhances endurance performance and aerobic characteristics more than high-volume training in trained rowers. Journal ofSports Sciences [Internet]. 2017 Jun [cited 2024 Aug 3];35(11):1052–8.Available from: https://search-ebscohost-com.ezproxy-prd.bodleian.ox.ac.uk/login.aspx?direct=true&AuthType=ip,shib&db=sph&AN=121549896&site=ehost-live	Study fails to report the correlation of the lactate score to 2000m rowing ergometer performance
Scott, A. T.,O'Leary, T., Walker, S., & Owen, R. (2015). Improvement of 2000-m Rowing Performance With Caffeinated Carbohydrate-Gel Ingestion. International Journal of Sports Physiology and Performance, 10(4), 464-468.	Study fails to report the correlation of the lactate score to 2000m rowing ergometer performance

De Campos Mello F,Bertuzzi R, Franchini E, Candau R. Rowing ergometer with the slide is more specific to rowers' physiological evaluation. RES SPORTS MED. . 2014;22(2):136-46.doi:10.1080/15438627.2014.881820	Study fails to report the correlation of the lactate score to 2000m rowing ergometer performance
Treff G, Schmidt W,Wachsmuth N, Volzke C, Steinacker JM. Total haemoglobin mass, maximal and submaximal power in elite rowers. Int J Sports Med. . 2014;35(7):571-4.doi:10.1055/s-0033-1358476	Study fails to report the correlation of the lactate score to 2000m rowing ergometer performance
Bourgois J, SteyaertA, Boone J. Physiological and anthropometric progression in an international oarsman: a 15-year case study. Int J Sports Physiol Perform. . 2014;9(4):723-6.doi:10.1123/ijspp.2013-0267	Study fails to report the correlation of the lactate score to 2000m rowing ergometer performance
Lawton TW, Cronin JB,McGuigan MR. Strength, power, and muscular endurance exercise and elite rowing ergometer performance. J Strength Cond Res. . 2013;27(7):1928-35.doi:10.1519/JSC.0b013e3182772f27	Study fails to report the correlation of the lactate score to 2000m rowing ergometer performance
Jurimae T,Perez-Turpin JA, Cortell-Tormo JM, et al. Relationship between rowing ergometer performance and physiological responses to upper and lower body exercises inrowers. J Sci Med Sport. . 2010;13(4):434-7.doi:10.1016/j.jsams.2009.06.003	Study fails to report the correlation of the lactate score to 2000m rowing ergometer performance
Driller MW, Fell JW,Gregory JR, Shing CM, Williams AD. The effects of high-intensity interval training in well-trained rowers. Int J Sports Physiol Perform. .2009;4(1):110-21. doi:10.1123/ijspp.4.1.110	Study fails to report the correlation of the lactate score to 2000m rowing ergometer performance
Bourdon PC, David AZ,Buckley JD. A single exercise test for assessing physiological and performance parameters in elite rowers: the 2-in-1 test. J Sci Med Sport. . 2009;12(1):205-11.doi:10.1016/j.jsams.2007.09.007	Study fails to report the correlation of the lactate score to 2000m rowing ergometer performance

Riganas CS, VrabasIS, Christoulas K, Mandroukas K. Specific inspiratory muscle training does notimprove performance or VO^sub 2max^ levels in well trained rowers. J Sports MedPhys Fitness 2008 09;48(3):285-92.	Study fails to report the correlation of the lactate score to 2000m rowing ergometer performance
Ingham SA, Carter H,Whyte GP, Doust JH. Physiological and performance effects of low- versus mixed-intensity rowing training. Med Sci Sports Exerc. . 2008;40(3):579-84.doi:10.1249/MSS.0b013e31815ecc6a	Study fails to report the correlation of the lactate score to 2000m rowing ergometer performance
Perkins CD, Pivarnik JM. Physiological Profiles and Performance Predictors of a Women's NCAA Rowing Team. Vol. 17, National Strength & Conditioning Association J. Strength Cond. Res. 2003.	Study fails to report the correlation of the lactate score to 2000m rowing ergometer performance
EMBASE SEARCH	
Jurimae J., JurimaeT., Maestu J., Pihl E. Prediction of 2000-m rowing performance on single sculls from metabolic and anthropometric variables. J. Sports Sci. 2000;18(7):514-515.	Study fails to report the correlation of the lactate score to 2000m rowing ergometer performance
Russell A.P., LeRossignol P.F., Sparrow W.A. Prediction of elite schoolboy 2000-m rowing ergometer performance from metabolic, anthropometric and strength variables. J.Sports Sci. 1998;16(8):749-754. doi:10.1080/026404198366380	No submaximal Lactate Tests or maximal blood lactate concentration or maximal plasma lactate concentration
