# Supplementary material for: Associations Between Lactate Thresholds and 2000 m Rowing Ergometer Performance: Implications for Prediction—A Systematic Review
Source: Sports Med Open. 2025 Feb 28;11:21. doi: 10.1186/s40798-024-00796-4 (PMC11871166; doi:10.1186/s40798-024-00796-4)
Supplement: Supplementary file 2 — Additional file 2. Online Resource 2 - Data Extraction. [file 40798_2024_796_MOESM2_ESM.pdf]

Title: Utility of lactate testing for the prediction of 2000m rowing ergometer performance: a systematic review

Journal: Sports Medicine – Open

Author Names: Timothy Kilbey†, Eugenio Vecchi†, Paulo Salbany, Prof. Ashok Handa, Prof. Eleanor Stride, Mihir Sheth*

† These authors contributed equally to this work.

*Correspondence should be addressed to mihir.sheth@nds.ox.ac.uk

Affiliations

Department of Engineering Science, Institute of Biomedical Engineering, University of Oxford, Oxford, U.K

Mihir Sheth, Prof. Eleanor Stride

Nuffield Department of Surgical Sciences, University of Oxford, Oxford, U.K

Prof. Ashok Handa, Paulo Salbany

St Catherine's College, University of Oxford, Oxford, U.K

Timothy Kilbey, Eugenio Vecchi

Online Resource 2: Data Extraction

Where possible, the following data from each study was extracted:

1) Authors	10) S.D of participant ages	19) Range of 2000m ergometer times
2) Study Title	11) Category of rower	20) Mean and S.D ergometer power over 2000m
3) Journal Name	12) Standard of Rowers	21) Range of 2000m ergometer power scores
4) Journal Volume	13) Results of lactate tests(s)	22) Mean and S.D ergometer speed over 2000m
5) Page Numbers	14) Correlation and significance of lactate test(s) to 2000m time/speed/power	23) Range of 2000m ergometer speeds
6) Year of Publication	15) Method of calculating LT	24) Type of correlation calculation
7) Number of participants	16) Methods of generating other lactate variable data	25) Type and results of statistical test performed
8) % Male participants	17) Other measurements used to predict performance	
9) Mean age of participants	18) Mean and S.D of ergometer time over 2000m	
