# Supplementary material for: Associations Between Lactate Thresholds and 2000 m Rowing Ergometer Performance: Implications for Prediction—A Systematic Review
Source: Sports Med Open. 2025 Feb 28;11:21. doi: 10.1186/s40798-024-00796-4 (PMC11871166; doi:10.1186/s40798-024-00796-4)
Supplement: Supplementary file 3 — Additional file 3. Online Resource 3 - Protocol. [file 40798_2024_796_MOESM3_ESM.pdf]

Title: Utility of lactate testing for the prediction of 2000m rowing ergometer performance: a systematic review

Journal: Sports Medicine – Open

Author Names: Timothy Kilbey†, Eugenio Vecchi†, Paulo Salbany, Prof. Ashok Handa, Prof. Eleanor Stride, Mihir Sheth*

† These authors contributed equally to this work.

*Correspondence should be addressed to mihir.sheth@nds.ox.ac.uk

Affiliations

Department of Engineering Science, Institute of Biomedical Engineering, University of Oxford, Oxford, U.K

Mihir Sheth, Prof. Eleanor Stride

Nuffield Department of Surgical Sciences, University of Oxford, Oxford, U.K

Prof. Ashok Handa, Paulo Salbany

St Catherine's College, University of Oxford, Oxford, U.K

Timothy Kilbey, Eugenio Vecchi

Online Resource 3: Systematic Review Study Protocol

Protocol: Accuracy of lactate testing for the prediction of 2km rowing ergometer performance: a systematic review.

Introduction

Athletes and their coaches need to determine the effects of their training regime. However, a full race-distance maximal effort test is a physiological challenge that will interrupt an athlete's training and thus cannot be performed too frequently. This issue is even greater for marathon runners, who will compete in as little as two to three marathons per year, as each one necessitates an extended recovery period. As such, previous studies have investigated the remarkable strength of correlation between lactate tests and marathon times(1,2,3) However, other studies have concluded that lactate testing alone should not be used to predict trail running times(4). Nevertheless, lactate testing of rowers has become increasingly common as a way to determine their endurance capacity and monitor fitness. However, a 2km rowing race lasts only 5:30-7 min and thus the performance under anaerobic conditions may play a greater role than in marathon runners. In addition, there are a range of different lactate tests that are performed. This systematic review aims to assess the accuracy of various lactate tests for predicting rowing 2Km ergometer performance, as no recent, published systematic review tackles this question. The findings of this review may help enable better monitoring of a rower's athletic progress and the effects of training adaptations. It may also have wider consequences such as improving the ability of a coach to identify rowing performance potential in untrained athletes.

Objectives

1) To review and assess the extent, quality and reliability of lactate testing as a tool for predicting 2Km rowing ergometer times.

2) To assess whether this is an appropriate test for athletes to use as a substitute for the 2Km test to gain an idea of the efficacy of their training regime

Methods

Eligibility criteria

Studies that report the following will be eligible for inclusion in the review

- Individuals of any age or sex
- Amateur or professional rowers
- The use of submaximal (2mM or 4mM lactate thresholds) or maximal blood lactate concentration or maximal plasma lactate concentration
- Studies on rowing for 2Km
- Studies in English

We shall exclude

- Studies that fail to report the accuracy or reliability of the lactate scores and its correlation to the rowing performance

- Studies that do not use a rowing ergometer
- Studies not published in English

There will be no date restrictions for studies

Information Sources

- MEDLINE (ovid), EMBASE, SPORTdiscus.
- Google search
- The bibliography of included studies

Search Strategy

Example Medline strategy

search terms will be:

3) (lactate or anaerobic threshold* or aerobic threshold* or aerobic-anaerobic threshold* or anaerobic-aerobic threshold* or exercise test*)
4) (performance or time or fatigue or exhaustion or result*).mp.
5) (row* or ergo*).mp
6) (2Km or 2000m or 2,000m or two-thousand meter or two-thousand metre or two-kilomet* or 2-kilomet* or 2000-m or 2000 m or 2-Km).mp
7) 1 and 2 and 3 and 4

Selection Process

We shall screen records for eligibility based on title and abstract. The full texts of eligible studies will then be screened independently by two reviewers for inclusion in the review. Excluded studies will be documented, with reasons given. Any disagreements between the primary reviewers will be resolved through discussion with a third reviewer, who will also check the data for consistency and clarity.

Data Items

Authors

Study Title

Journal Name

Journal Volume

Page Numbers

Year of Publication

Number of participants

% male participants

Mean age of participants

Standard Deviation of participant ages

Category of rower

Standard of Rowers

Results of lactate tests(s)

Correlation of lactate test(s) to 2km time/speed/power and the significance of this correlation

Method of calculating lactate threshold

Methods of generating other lactate variable data

Other measurements used to predict performance

Mean and standard deviation of Ergometer time over 2km

Range of 2km ergometer times

Mean and standard deviation ergometer Power over 2km

Range of 2km power scores

Mean and Standard Deviation ergometer Speed over 2km

Range of 2Km speeds

Type of correlation calculation

Outcomes

The primary outcome will be the accuracy of the predicted ergometer time based on lactate testing data.

Risk of Bias in individual studies

The reviewers will independently assess the reporting quality and methodological quality of the included studies.

Results will be presented in a table, showing the correlation between lactate threshold and 2Km ergometer time or prediction accuracy for each study.

References

1. Sjödin, B., and I. Jacobs. "Onset of blood lactate accumulation and marathon running performance." *International journal of sports medicine* 2.01 (1981): 23-26.

2. Föhrenbach, R., A. Mader, and W. Hollmann. "Determination of endurance capacity and prediction of exercise intensities for training and competition in marathon runners." *International journal of sports medicine* 8.01 (1987): 11-18

3. Roecker, K., et al.. "Predicting competition performance in long-distance running by means of a treadmill test." *Medicine and science in sports and exercise* 30 (1998): 1552-1557.

4. de Waal, Simon J., et al. "Physiological indicators of trail running performance: a systematic review." *International Journal of Sports Physiology and Performance* 16.3 (2021): 325-332.
